# The Gamblification of Investing: How a New Generation of Investors Is Being Born to Lose

**DOI:** 10.3390/ijerph19095391

**Published:** 2022-04-28

**Authors:** Philip W. S. Newall, Leonardo Weiss-Cohen

**Affiliations:** 1 School of Psychological Science, University of Bristol, Bristol BS8 1TU, UK; 2 Department of Psychology, Kingston University, London KT1 2EE, UK; l.cohen@kingston.ac.uk

**Keywords:** trading, gambling, betting, financial markets, traders

## Abstract

Investing and gambling share key features, in that both involve risk, the coming together of two or more people, and both are voluntary activities. However, investing is generally a much better way than gambling for the average person to make long-run profits. This paper reviews evidence on two types of “gamblified” investment products where this advantage does not hold for investing: high-frequency stock trading and high-risk derivatives. This review defines a gamblified investment product as one that leads most investors to lose, that attracts people at risk of experiencing gambling-related harm, and that utilizes product design principles from gambling (either by encouraging a high frequency of use or by providing the allure of big lottery-like wins). The gamblification of investing produces novel challenges for the regulation of both financial markets and gambling.

## 1. Introduction

“Once in the dear dead days beyond recall, an out-of-town visitor was being shown the wonders of the New York financial district. When the party arrived at the Battery, one of his guides indicated some handsome ships riding at anchor. He said, ‘Look, those are the bankers’ and brokers’ yachts.’ ‘Where are the customers’ yachts?’ asked the naive visitor.”—Fred Schwed Jr. [1].

Schwed’s (1940) opening lines in a classic critique of Wall Street show how financial intermediaries have for a long time profited from their investor clients. Financial intermediaries—in this case bankers and brokers—pay for their yachts by charging commissions, fees, and spreads on every investment transaction made by their clients, as well as on-going charges on positions and balances. This aspect of exploitation in financial markets was later downplayed by post-war financial economists, who saw a world where pure “efficient” markets meant that all investors got fair returns in compensation for bearing risk [2]. In the most extreme “strong form” of the “efficient market hypothesis,” even company insiders were said to be unable to profit from their private knowledge, given the stock market’s perfect ability to form accurate prices [3]. One of the key underlying assumptions of the strong form of the efficient market hypothesis is that traders are fully rational and maximize their utilities. Modern behavioral economists have, however, observed numerous financial pricing anomalies, representing failures of the market to produce perfect prices, which provides evidence that the strong form of the efficient market hypothesis does not hold [4]. The literature on behavioral finance often blames such price anomalies on the irrational behavior of traders [5,6]. Some of the latest economic theorizing therefore accepts Schwed’s observation: that exploitation can occur in financial markets [7]. Instead of being fully rational, traders have “bounded rationality” constrained by limitations in cognitive capacity, and do not fully maximize their utilities—as a result, many behavioral biases have been observed [8]. For example, retail investors typically overspend, transferring large sums of wealth to intermediaries [9,10], even though higher expenditure does not translate into higher returns [11] or better services for investors [12]. This is similar to the “house edge” in gambling—the house always wins, while taking minimal to no risk [13]. In both domains, while large gains are unpredictable and unlikely, fees are guaranteed. This article reviews how an emerging “gamblification” of financial markets is the latest iteration of this exploitation of personal investors.

Investing and gambling share certain key features. In fact, they are similar enough that some financial economists have tested their theories on sports betting markets, as an analogous yet simpler version of the stock market [14,15]. Both involve risk the coming together of two or more people, are voluntary activities [16], and are motivated by financial gains [17]. However, perhaps the biggest difference is that the stock market allows for a greater potential for ordinary people to earn long-run profits [16]. Selling stock allows companies to invest in profitable ventures, and a share of these earnings get returned to investors, allowing everyone who owns a share in the company to profit over time [18]. This makes the stock market a “positive-sum game” in the words of economists, where everyone who plays can profit. Gambling, meanwhile, does not lead to the creation of new wealth, merely the redistribution of money wagered, and is therefore a “zero-sum game.” Gambling games can then be split into two broad categories. First, “non-skilled” gambling games, such as roulette or slots, ensure long-run losses for gamblers, as any potential payoffs do not provide sufficient return for their likelihood of occurring [19]. Second, “skill-based” gambling games, such as sports betting or poker, can allow for some gamblers to win in the long run [20,21]. However, this profitability is not readily available to the average gambler, as nowadays professional gamblers resemble hedge funds in their use of large datasets and artificial intelligence [22,23]. This information largely stays within small expert communities, who guard their superior knowledge against outsiders in order to maintain their future profitability [24]. Investing, therefore, can offer the average person the possibility of long-run profits, while gambling cannot.

A “gamblified” investment product is here defined as having three key features. First, it must resemble gambling in being designed to reduce the proportion of investors using it who earn long-run profits. Second, it must elicit from investors who lose money similar behavior patterns as to those found in gamblers who experience harm. Third, the case for gamblification is strengthened if the product uses key design principles first honed by the gambling industry to create unprofitable yet alluring products [25,26]. 

As with the wide range of gambling games, there are also many different investment products, with various differences existing across countries and a significant amount of ongoing product innovation. This makes it impossible for any review to give due consideration to all investment products that could conceivably meet the definition of being gamblified. Therefore, this review will focus only on the two types of investing that have the strongest amount of evidence for meeting this three-part definition of gamblification: high-frequency stock trading and high-risk derivatives, such as options, futures, and contracts-for-difference (greater clarification of these terms is provided below). It is hoped that this review can help guide further research into other investment products that cannot be covered here.

It is important to highlight that this is a subset of all the investment products available, and that not all investment products are being gamblified: most (sensible) investment products have long-term benefits for investors. Mutual funds, for example, can enable individual investors to easily reap the risk-reduction benefits from a diversified portfolio [27]. “Index” mutual funds are an especially beneficial product, and have since the 1970s allowed personal investors to easily invest in broad baskets of stocks, such as the Dow Jones Index, at very low fees [28]. Index funds’ low fees reduce the proportion of money going to financial intermediaries, thereby allowing personal investors to capture most of the long-run growth from companies [18]. Importantly, index funds make limited trades, which leads to fewer commissions for intermediaries, and avoid attempting to beat Wall Street intermediaries at predicting stock price movements. This makes index funds an innovation that Schwed (1940) would likely have approved of. Despite the existence of many sensible approaches to investing, recent years have also seen rapid growth in investment products that could be considered to be gamblified.

Problem gambling is associated with a number of harms, and these harms can occur well before an individual reaches the threshold for “problem gambling” on a self-report scale [29,30]. Recently, the COVID-19 pandemic and its lockdowns have been associated with an observed increase in online gambling as a result of increased anxiety, depression, and reduced working hours [31]. In particular, gamblers turned to online platforms due to restrictions on physical gambling venues, which were closed as a result of lockdowns [32]. The pandemic has also led to an increase in online retail investments, both with existing investors trading more intensively and new clients opening new accounts [33]. In many jurisdictions, online gambling is heavily regulated, and therefore it is not surprising that many individuals have turned to online investments instead, and that providers have exploited this trend by further “gamblifying” their products.

The below sections outline how high-frequency stock trading and high-risk derivatives meet the three-part definition of being gamblified.

## 2. High-Frequency Stock Trading

### 2.1. Why High-Frequency Stock Trading Is a Losing Strategy

The two main strands of financial economics both recommend that the average investor should rarely buy and sell stocks, known collectively as “trading.” The efficient market view states that any stock that an investor sells is likely to be about as good as the stock that is bought to replace it [3]. Given this fact, the uncertain gains of trading are unlikely to outweigh the certain costs of trading, which include direct brokerage commissions, payment of the bid-ask spread, and potential taxes [34]. The behavioral finance view also acknowledges all of these downsides of trading, and adds to these the fact that many investors tend to make fundamentally bad trades, where the stock bought often does less well than the stock that was sold [35]. As a result, empirical research has consistently shown that higher frequencies of stock trading are associated with worse returns. One study using data on personal investors from the US in the early 1990s showed that the most frequent traders earned on average 5% less per year than the average investor, who still turned over a likely excessive 75% of their portfolio annually [34]. 

However, these returns are still higher than those of the highest-frequency stock traders. “Day trading” is considered to be the highest-frequency form of stock trading amongst personal investors, and is defined as the buying and selling of the same stock in a single day. One of the consistent findings from historical stock price data is that, on average and over the long term (e.g., years), stocks provide consistently positive returns [27]. However, prices are subject to unpredictable noises and irrational behavioral biases in the short term, making prices random and impossible to predict within the ultra-short time-frames of a day trader. Because of this increase in volatility, a dataset covering a 12-year period of investor returns concluded that only around 5% of day traders are profitable [36]. This extreme form of high-frequency stock trading meets at least the first aspect of gamblification, in that the vast majority of those investors lose money over time. 

### 2.2. Behavioral Similarities with Gambling

People experiencing gambling-related harm usually show signs of behavioral dependence, in that they spend more time engaging in gambling and thinking about gambling than they would prefer [37]. Similar patterns have also been observed amongst a group of “problematic” investors [38,39]. Frequent trading and gambling can both be attributed to the same personality traits, such as overconfidence, impulsiveness and sensation seeking [40,41,42]. Online trading, a recent trend increasing in particular during the recent pandemic, has been suggested to boost confidence (in comparison with offline trading) and lead to more intensive trading [43]. A number of international studies have observed that a small proportion of investors refer themselves to gambling treatment clinics to deal with the consequences of their investing behavior [44,45,46,47]. More broadly, given that only a small minority of gamblers refer themselves to treatment clinics, a machine-learning analysis of text from a gambling self-support online forum found that investing topics formed one of the ten most prominent themes emergent from the data [48]. “Problem investing” scales can also be constructed that closely follow instruments used for problem gambling, and have been shown to correlate with self-reported risky investing behavior [49,50]. Furthermore, a study of 61 day-traders showed that they were much more highly engaged with gambling activities than a larger group of investors who traded less frequently [51].

However, these studies either rely on small numbers of participants referring themselves to clinics who might be only a small minority of investors, or use novel investing scales, which may have been less stringently validated than scales used in gambling research [52]. One study showed that higher rates of stock trading were associated with problem gambling scores across a sample of 795 personal investors from the US [53]. This suggests that trading too frequently might be problematic for more than just the most highly engaged investors. Collectively, the research reviewed in this section shows a number of behavioral similarities between gambling and frequent stock trading.

### 2.3. Product Design Principles from Gambling

Some gambling products are used more commonly by people experiencing gambling-related harm than others. One recurring attribute of these harmful gambling products is the ease with which they facilitate gambling repeatedly in short spaces of time [54,55]. Internationally, electronic gambling machines were perhaps the first product to become widely known for this attribute and association [25]. However, similar observations have since been made for high-frequency sports betting products [56,57], online gambling [58], and mobile gambling [59]. All of these innovations in gambling product design have increased the potential frequency with which people can gamble.

Similarly, a new generation of mobile investing apps is making it easier than ever before to invest actively in the stock market [60]. The ability to trade anywhere via mobile likely increases investors’ propensity to trade in comparison to online- or phone-based trading. These mobile investing apps frequently market themselves through elite sport, a marketing strategy first developed by the gambling industry [61]. This marketing often emphasizes that the apps have either low or zero explicit fees [60], and the apps themselves can contain constant notifications and gamification elements nudging investors toward more frequent trading [62]. These apps can even profit from charging zero explicit fees via a financial arrangement called “payment for order flow” (Rooney & Fitzgerald, 2020) [63]. This extent to which financial trading apps do not clearly communicate the true cost of their use is similar to the ways gambling operators obfuscate mandated cost-of-play information on gambling products [64,65].

## 3. High-Risk Derivatives

### 3.1. Why Investing in High-Risk Derivatives Is a Losing Strategy

This section focuses on the financial products of options, futures, contracts-for-difference (collective known as derivatives), and foreign exchange speculation via high-risk derivatives. These are all products that provide a payoff based on the price of another underlying financial asset, and so can be used to reduce risks for actors with certain liabilities via “hedging” [66]. For example, a wheat farmer can use a future on the price of wheat to guarantee a given income from their end-of-year harvest. However, if traded separately from their underlying financial asset (i.e., without the purpose of hedging), these products can also be used speculatively as risky bets with small chances of winning large amounts of money [67]. Options can be used to magnify the wins from a stock’s price increase compared to the gain from buying the stock, or can even be used to profit from a fall in the price (i.e.,” short selling”), such as by “buying a put option” [68]. Futures can be used to dramatically increase an investor’s financial exposure to market movements (i.e., “leveraging”), as no initial financial outlay is required, apart from any collateral held to cover for potential losses. Contracts for difference (CFDs) and leverage certificates (LCs) can perform very similar goals for investors, with the main difference being between jurisdictions, with options and futures being favored in the US and contracts for difference favored elsewhere, due to differences in taxation and in the ways contracts are settled [69]. Foreign exchange speculation involves betting on the price movements of currencies, often via the use of derivatives.

These high-risk derivatives are often difficult to understand and have complicated non-linear payment structures, and because their prices are dependent on the underlying assets, which move stochastically, they are notoriously difficult to value. Research suggests that most personal investors lose money overall in these markets, due to lack of understanding by unsophisticated investors of the complexity and risks associated with them. A study from Brazil suggested that 97% of futures traders suffer annual losses [70], which is similar to the rates suffered by stock day traders. Data presented by the US Securities and Exchange Commission suggest that foreign exchange traders usually lose all of their money within a year [71]. In the Netherlands, a study on options trading by retail investors concluded that most individuals incur substantial losses, with an average monthly negative return of −1.81% [72]. Profitability analysis of Germans trading leverage certificates of single stocks (which are structurally similar to CFDs) generated negative returns between −4.96% and −9.45% (for comparison, in the same period, the German stock index went up 30%); these products were also highly leveraged, with an average nine-fold increase in volatility and risk-taking, with every 1% move in asset prices translating into a 9% gain or loss for the investor [73]. High amounts of leverage have also been blamed for the losses experienced by more than 80% of clients trading CFDs [74]. Another study utilizing the social data provided by a popular contract-for-difference trader shows that around 80% of traders lose money annually [71], while disclosures from the EU’s financial regulator suggest that between 74% and 89% of traders lose money annually [75]. Although some traders will win money in these markets, and because of the inherent riskiness, some of these wins may be significant, these data are consistent with gamblification in that the average investor loses money on these products.

### 3.2. Behavioral Similarities with Gambling

Previous evidence also points towards similarities between engagement with various high-risk derivatives and gambling. For example, studies have assessed participants’ self-reported engagement with, “high-risk stocks, options, futures or day trading” [76,77]. Although this definition of speculative investments is rather broad, these studies did find that rates of engagement with these types of investments were elevated amongst participants experiencing high rates of gambling-related harm. By comparison, another study asked about engagement with these investments separately, while also getting participants to answer a “problem investing” questionnaire adapted from gambling [50]. Results of this study showed for example that “problem investors” were two times more likely to invest in options than non-problematic investors. These studies provide evidence for behavioral similarities between gambling and the use of high-risk derivatives.

### 3.3. Product Design Principles from Gambling

High-risk derivatives also have certain design features that are analogous to gambling. Although these products were initially only readily available for sophisticated high-net-worth investors, it has recently been increasingly easy for personal investors to use them via mobile investing apps [60]. Compared to stock trades, option trades can yield especially high payments for order flow to trading apps [63], providing a financial incentive to make these complex investments more readily available to investors.

Providing investors with the allure of big potential wins is the main product design feature adapted from gambling into high-risk derivatives [78]. Lotteries are perhaps the most well-known gambling product selling hope in this way [79], but this feature has also been adopted by other gambling products, including electronic gambling machines [80] and sports betting products [81]. However, the data from sports betting suggest that these high payoffs occur very rarely. This trend toward offering high potential wins across a range of gambling products is relevant because research shows that gamblers experiencing high levels of harm are the most likely to seek out these high potential wins [82]. The perceived need for these gamblers to recoup losses may be one reason for this preference [83,84]. Overall, this consistency across gambling and investing of product design tending towards providing the hope of big potential wins is also consistent with the gamblification of investing.

The allure of very rare but potentially big wins can potentially be exacerbated by social media via survivorship bias. Those rare individuals who win big sums in gambling or investing will advertise their (random and not replicable or sustainable) high-risk strategies using social platforms, which are likely to be shared and promoted extensively. Such advertised strategies are likely to be unsuccessfully followed, as people try to replicate other people’s behavior [85]. Conversely, people who lose are less likely to advertise their failures, and even if they do, are less likely to attract attention. This promotes a biased view that high-risk trading and gambling lead to fortunes [86].

## 4. Conclusions

Aspects of investing have always been consistent with a small group of financial intermediaries profiting at the expense of personal investors [1]. Some investment products are more favorable toward personal investors, and the growth in index funds is one example of the uptake of a beneficial investment product. However, this review argues that a recent trend toward “gamblified” investment products is a new way for financial intermediaries to profit at the expense of personal investors. A gamblified investment product is here defined as one that leads most investors to lose, that attracts people at risk of experiencing gambling-related harm, and that utilizes product design principles from gambling. Evidence was, thus, reviewed in support of high-frequency stock trading and high-risk derivatives meeting this definition. Although these are only two product categories, the structure of this review could potentially guide future research into the potential gamblification of other investment products, such as, for example, cryptocurrencies.

Cryptocurrencies were deemed beyond the scope of this review, as unlike stocks and derivatives, they did not originate from the traditional financial industry. Unlike stocks, cryptocurrencies do not generally provide dividends, making them less of a traditional investment. In addition, cryptocurrencies are more volatile than fiat currencies, introducing another difference from foreign exchange trading. In addition, the evidence base on gambling and cryptocurrency was deemed as being less mature than the evidence surveyed here. Studies have, however, found links between cryptocurrency use and problem gambling [87,88], suggesting that this may be a fruitful area for further investigation. Furthermore, cryptocurrencies may also involve different psychological factors to those at play in more traditional areas of investment [89].

In the domains of trading and investments, there are increased rewards for increased risk taking. In investments, this is known as the “risk premium”, and investors often demand higher returns for riskier investments [18]. The same occurs in gambling: betting on a longshot (higher risk) is associated with higher payouts. However, there is a crucial difference between the two domains. Financial investors appear to be risk-averse, demanding higher expected returns for risky investments than deemed fair by economic theory, a phenomenon that has been named the “equity premium puzzle” [90]. Conversely, gamblers appear to be risk-seeking, often accepting much lower expected returns for longshot bets that should pay considerably more in order to be fair [15,65]. One of the problems of the gamblification of investing products is perhaps the bringing of this risk-taking gambler mentality into the stock markets, which in turn translates into apparent irrational behavior, such as high-frequency trading and betting on high-risk derivatives.

The gamblification of investing poses issues for financial regulators. The US financial regulator recently announced that it is considering whether to regulate the features of mobile investing apps [91]. As argued earlier, the regulation of these apps’ features can be informed by the gambling literature on structural characteristics. Some financial regulators have begun to consider more broadly the fact that many personal investors are not as rational as assumed in traditional economics [92]. It may be the case that financial regulators can learn from gambling regulators and the gambling literature about how to regulate these products.

Finally, the gamblification of investing also poses issues for regulators’ attempts to reduce gambling-related harm. Certain jurisdictions, such as the UK, are currently restricting rules around gambling marketing and products [93]. However, the proliferation of gamblified investment products is a topic outside of the scope of any current changes, and could undermine attempts to reduce gambling-related harm. For example, people who have experienced gambling related-harm and who, for example self-excluded themselves from being able to gamble [94], can nonetheless still access gamblified investment products and be exposed to their marketing. Most people perceive “investing” as being less risky than gambling [95], and sensible investing does provide sensible positive returns; however, the gamblification of investing is making trading unnecessarily harmful, while helping finance those bankers’ yachts.
